# Successful Treatment of Hailey-Hailey Disease With Dupilumab

**DOI:** 10.7759/cureus.86817

**Published:** 2025-06-26

**Authors:** Jake Breimann, Saira N Agarwala, Shayan Waseh, Sylvia Hsu

**Affiliations:** 1 Dermatology, Temple University Hospital, Philadelphia, USA

**Keywords:** acantholytic dermatosis, dupilumab, familial benign pemphigus, hailey-hailey disease, monoclonal antibody

## Abstract

Hailey-Hailey disease (HHD) is a rare autosomal dominant genodermatosis caused by mutations in the ATP2C1 gene, leading to impaired calcium homeostasis and epidermal acantholysis. Clinically, it manifests as recurrent, painful erosions in the intertriginous areas and is often resistant to conventional treatments, such as topical corticosteroids, antibiotics, and retinoids. This report describes the case of a 67-year-old woman with refractory HHD who presented with painful, pruritic erosions affecting the axillary, inguinal, and inframammary regions. Despite prior treatment with high-potency topical corticosteroids and oral retinoids, her symptoms persisted. Given the chronic and relapsing nature of HHD, dupilumab, an IL-4/IL-13 inhibitor, was initiated. The patient experienced significant symptomatic improvement within two months of therapy. This case contributes to the growing body of evidence supporting dupilumab as a potential therapeutic option for HHD and highlights the need for further research to evaluate its long-term efficacy and safety in managing this condition.

## Introduction

Familial benign pemphigus, or Hailey-Hailey disease (HHD), is an autosomal dominant genodermatosis characterized by recurrent vesiculobullous eruptions in the intertriginous areas. It is caused by mutations in the ATP2C1 gene, which disrupt calcium-dependent signaling and impair desmosomal and adherens junction integrity [1]. Clinically, HHD follows a chronic, relapsing-remitting course, with fragile vesicles and bullae that rupture to form painful erosions. Current therapeutic options, including topical corticosteroids, antibiotics, carbon dioxide laser ablation, and dermabrasion, focus primarily on managing exacerbations [2]. Here, we report a case of HHD successfully treated with dupilumab.

## Case presentation

**Figure 1:**
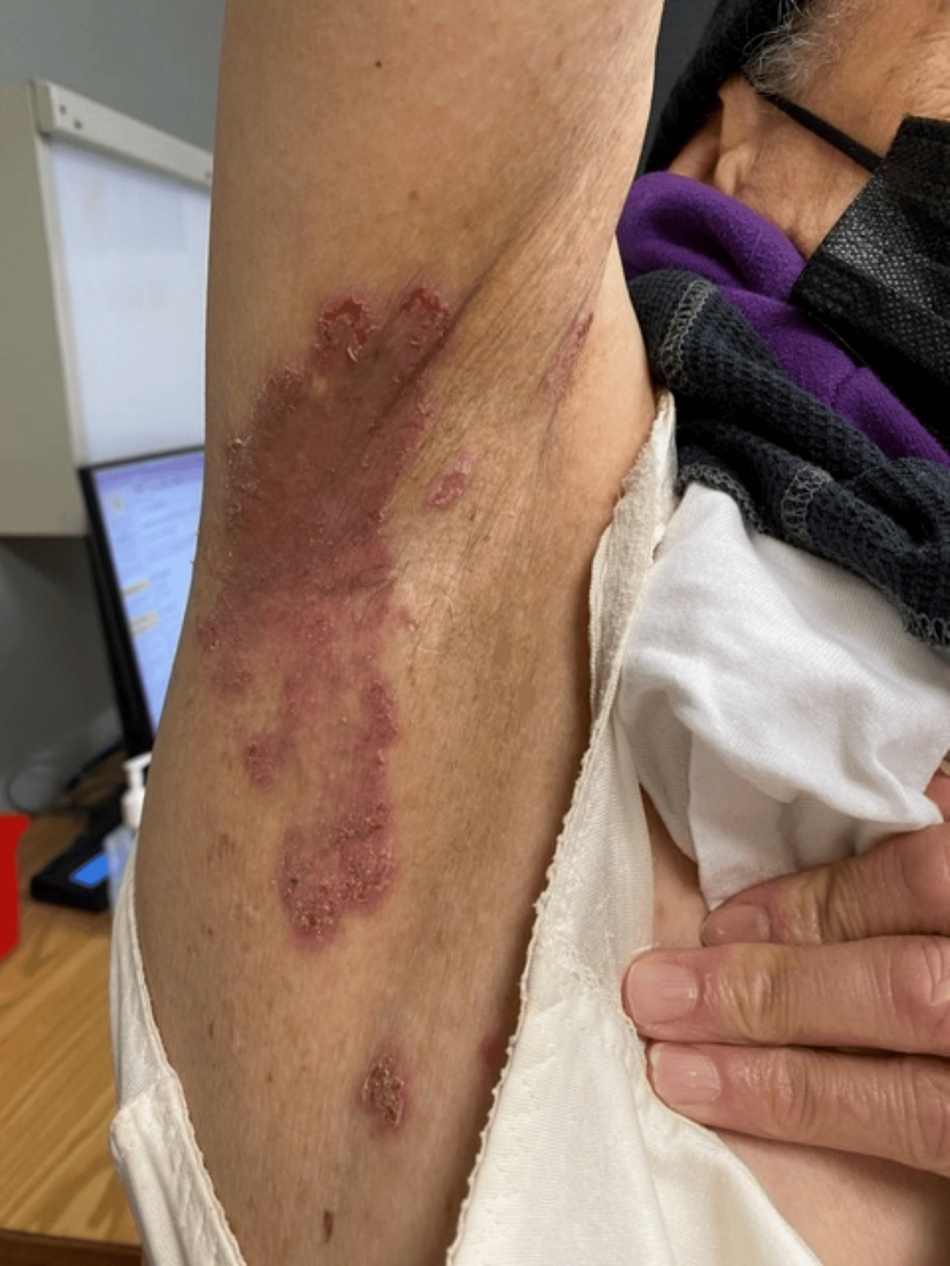
Hailey-Hailey disease in the right axilla.

**Figure 2:**
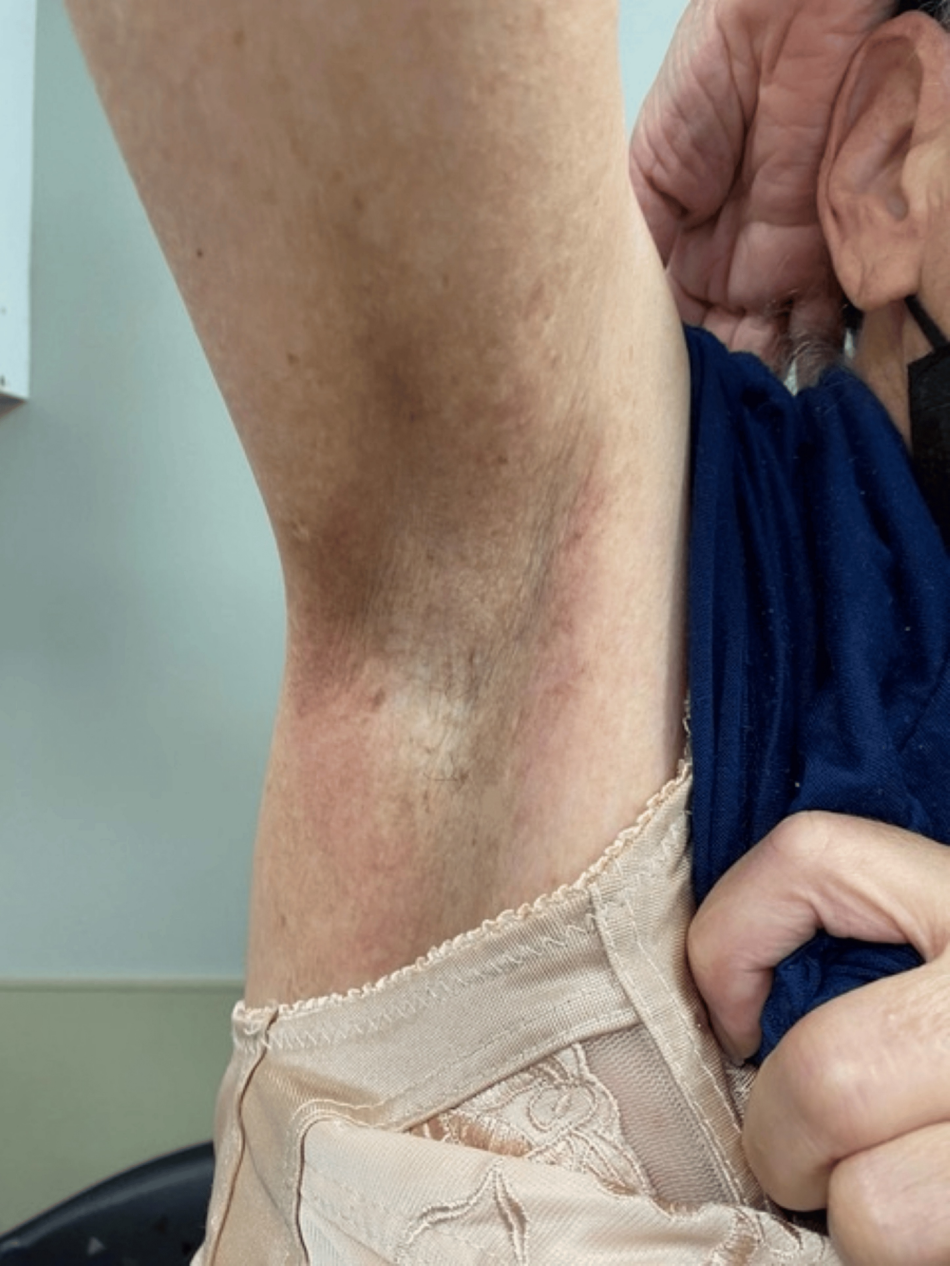
Clinical clearance of Hailey-Hailey disease in the right axilla after two months of dupilumab treatment.

A 67-year-old woman with a medical history of primary biliary cirrhosis, sickle cell trait, prediabetes, and HHD, previously diagnosed via skin biopsy at an outside institution in 2008, presented to our dermatology clinic for further management. She reported a more than 40-year history of symptomatic flares and noted that several paternal relatives had biopsy-confirmed HHD. Given the characteristic clinical features, positive family history, and prior histopathologic confirmation, a repeat biopsy was deemed unnecessary. At the time of presentation, she was being treated by outside dermatologists with topical mupirocin and fluocinonide, but without sustained benefit. Over a five-year period in our clinic, she was received with multiple courses of high-potency topical corticosteroids and oral retinoids, including acitretin and isotretinoin, during disease flares. However, these therapies provided only limited control. Immediately prior to initiating dupilumab, she presented with erosive, painful, and pruritic lesions** **in the axillary (Figure 1), inguinal, and inframammary regions, with symptom exacerbation often occurring during the summer months. Dupilumab was initiated using the standard regimen for atopic dermatitis (600 mg subcutaneous loading dose, followed by 300 mg subcutaneously every two weeks). At her two-month follow-up, she reported marked symptomatic improvement, and physical examination revealed substantial resolution of the lesions (Figure 2).

## Discussion

Currently, the literature on the use of dupilumab for HHD is limited, with only a few published case reports and case series [3]. Dupilumab is a monoclonal antibody that targets the interleukin-4 receptor alpha subunit, thereby inhibiting the activity of both IL-4 and IL-13, which share this receptor component. These cytokines are key mediators in type 2 inflammatory immune responses. A recent study demonstrated that IL-4 and IL-13 downregulate the expression of SPARC-related modular calcium-binding protein 1 (SMOC1) in keratinocytes. SMOC1 plays a pivotal role in regulating calcium influx in response to extracellular calcium concentrations, and its suppression leads to reduced intracellular calcium signaling and diminished expression of keratinocyte differentiation markers [4]. In the pathogenesis of HHD, mutations in the ATP2C1 gene, encoding a calcium-dependent ATPase, impair calcium transport into the Golgi apparatus, resulting in a functional calcium deficiency. Keratinocytes are particularly sensitive to this dysregulation, which contributes to acantholysis and impaired epidermal integrity [5]. Dupilumab may exert therapeutic benefit in HHD by reversing IL-4- and IL-13-mediated suppression of SMOC1, thereby partially restoring calcium-dependent keratinocyte differentiation and function.

## Conclusions

This case adds to the growing body of evidence supporting dupilumab as a potential treatment option for refractory HHD, offering a novel therapeutic approach for patients unresponsive to conventional therapies. By targeting IL-4 and IL-13, dupilumab may help mitigate the calcium dysregulation central to the pathogenesis of HHD. The significant clinical improvement observed in this patient highlights the need for further research to evaluate the long-term efficacy, safety, and optimal role of dupilumab in HHD management. Larger case series and controlled clinical trials are essential to determine whether dupilumab can provide sustained disease control, reduce flare frequency, and potentially modify the disease course. Expanding research into cytokine-targeted therapies may help refine treatment strategies and ultimately improve outcomes for patients living with this challenging condition.
